# Prolactin Changes as a Consequence of Chemical Exposure

**DOI:** 10.1289/ehp.114-a573

**Published:** 2006-10

**Authors:** Lorenzo Alessio, Roberto Lucchini

**Affiliations:** Institute of Occupational Health, University of Brescia, Brescia, Italy, E-mail: lucchini@med.unibs.it

We read with great interest the article by de Burbure et al. (2006) on health effects in children who live near nonferrous smelters in France, the Czech Republic, and Poland. We were especially interested in the inverse relationship found between levels of urinary mercury and serum prolactin. We found a similar result in an Italian multicenter crosssectional survey with adult subjects (Alessio et al. 2002) using a different statistical approach based on regression analysis with mixed linear models. We found that serum prolactin decreased as a function of both urinary mercury and occupational exposure to inorganic mercury (Lucchini et al. 2003). In another study (Carta et al. 2003), our group observed the opposite behavior of prolactin in adult individuals with a high dietary intake of mercury-contaminated tuna. In that study, serum prolactin was positively associated with urinary and blood mercury. Our interpretation of this dual behavior was that prolactin may be differently affected by inorganic and organic mercury based on the interference with different neurotransmitters implicated in the regulation of prolactin secretion (Carta et al. 2003).

The article by de Burbure et al. (2006) stimulates futher consideration of the observed effects on serum prolactin after exposure to various metals and other chemical substances. In fact, prolactin can be increased by exposure to lead (Govoni et al. 1987; Lucchini et al. 2000), organic mercury (Carta et al. 2003), and manganese (Ellingsen et al. 2003; Smargiassi and Mutti 1999; Takser et al. 2004), but it can be decreased by exposure to inorganic mercury (de Burbure et al. 2006; Lucchini et al. 2003; Ramalingam et al. 2003), alluminum (Alessio et al. 1989), and cadmium (Calderoni et al. 2005; de Burbure et al. 2006). Subjects exposed to chemicals such as styrene (Bergamaschi et al. 1996; Luderer et al. 2004; Umemura et al. 2005), perchloroethylene (Beliles 2002; Ferroni 1992), and anesthetic gases (Lucchini et al. 1996; (Marana et al. 2003) have shown an increase of serum prolactin, whereas polychlorinated biphenyls (De Krey et al. 1994) and the pesticide lutheinate [U.S. Environmental Protection Agency (EPA) 2002] are known to decrease serum prolactin.

Possible mechanisms, other than direct effects at the cellular level, may be related to different neurotransmitters involved in the modulation of prolactin secretion. For example, the dopaminergic and serotoninergic systems, respectively, are involved in the physiologic regulation of this hormone as a tonic inhibitor and as an excitatory modulator. Different chemicals may interfere with these two systems, resulting in different outcomes regarding serum prolactin. Recent studies have shown that the same chemical may even cause different effects on prolactin depending on the exposure doses (Lafuente et al. 2003).

We would like to know why this neuro-endocrine hormone is affected differently by exposure to different chemicals. This is important because of the possible use of prolactin, as described by de Burbure et al. (2006), as a sensitive indicator of early effects in toxicologic research and risk assessment (Mutti and Smargiassi 1998). Negative studies have also been published on the association of prolactin with the exposure to neurotoxicants (Myers et al. 2003; Roels et al. 1992). Therefore, it is vital to assess the causes of the variability that may limit the reproducibility of these tests. Further research should focus on multiple exposure to different chemicals, which may help to explain the lack of association.
